# Supplementary figures and images for: Transcriptional regulation of SARS-CoV-2 receptor ACE2 by SP1
Source: eLife. 2024 Feb 20;13:e85985. doi: 10.7554/eLife.85985 (PMC10878691; doi:10.7554/eLife.85985)

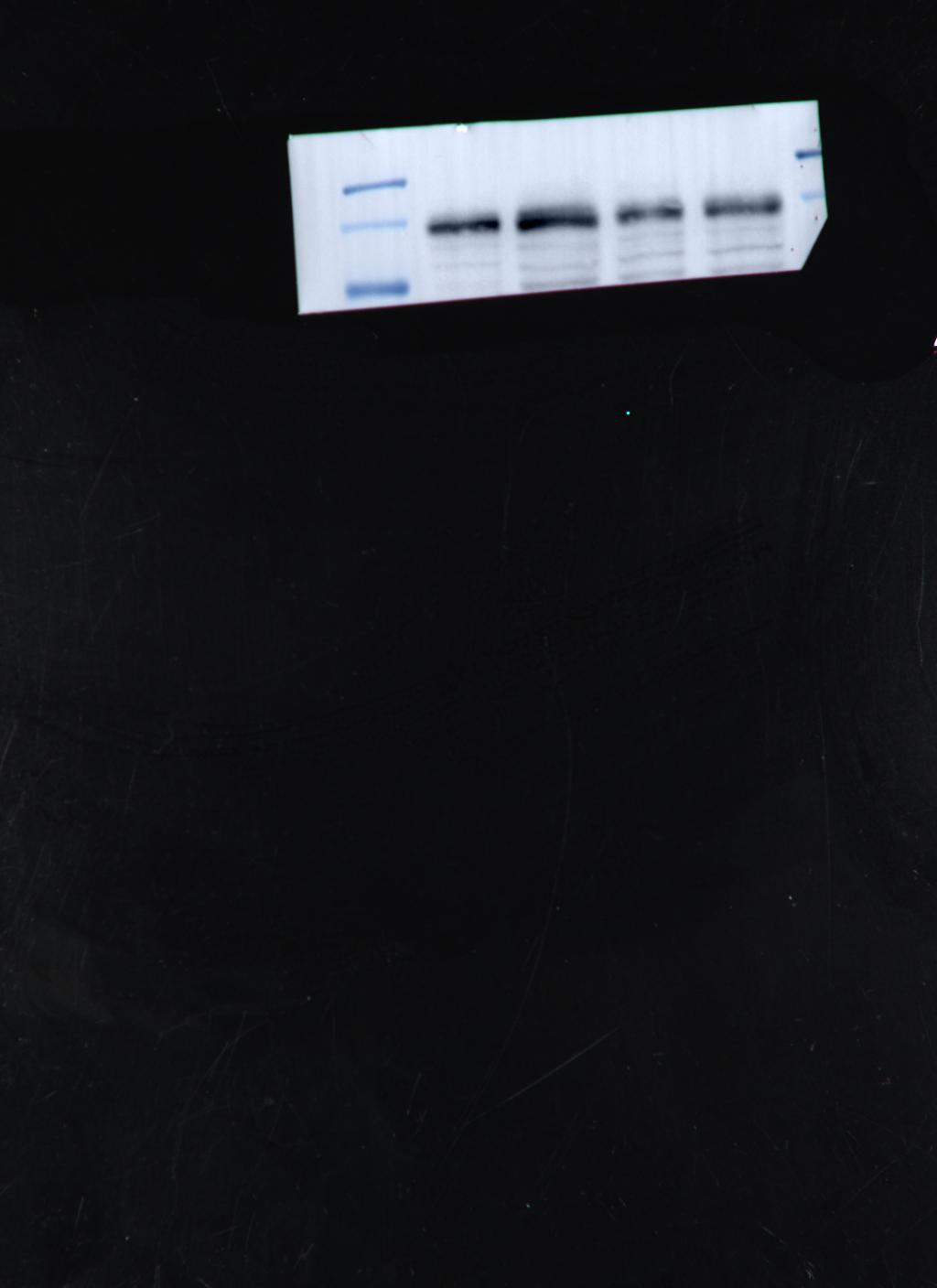

Supplement: Figure 1—source data 1. [file elife-85985-fig1-data1.zip › Figure 1-source data 1/anti-ACE2.jpg]

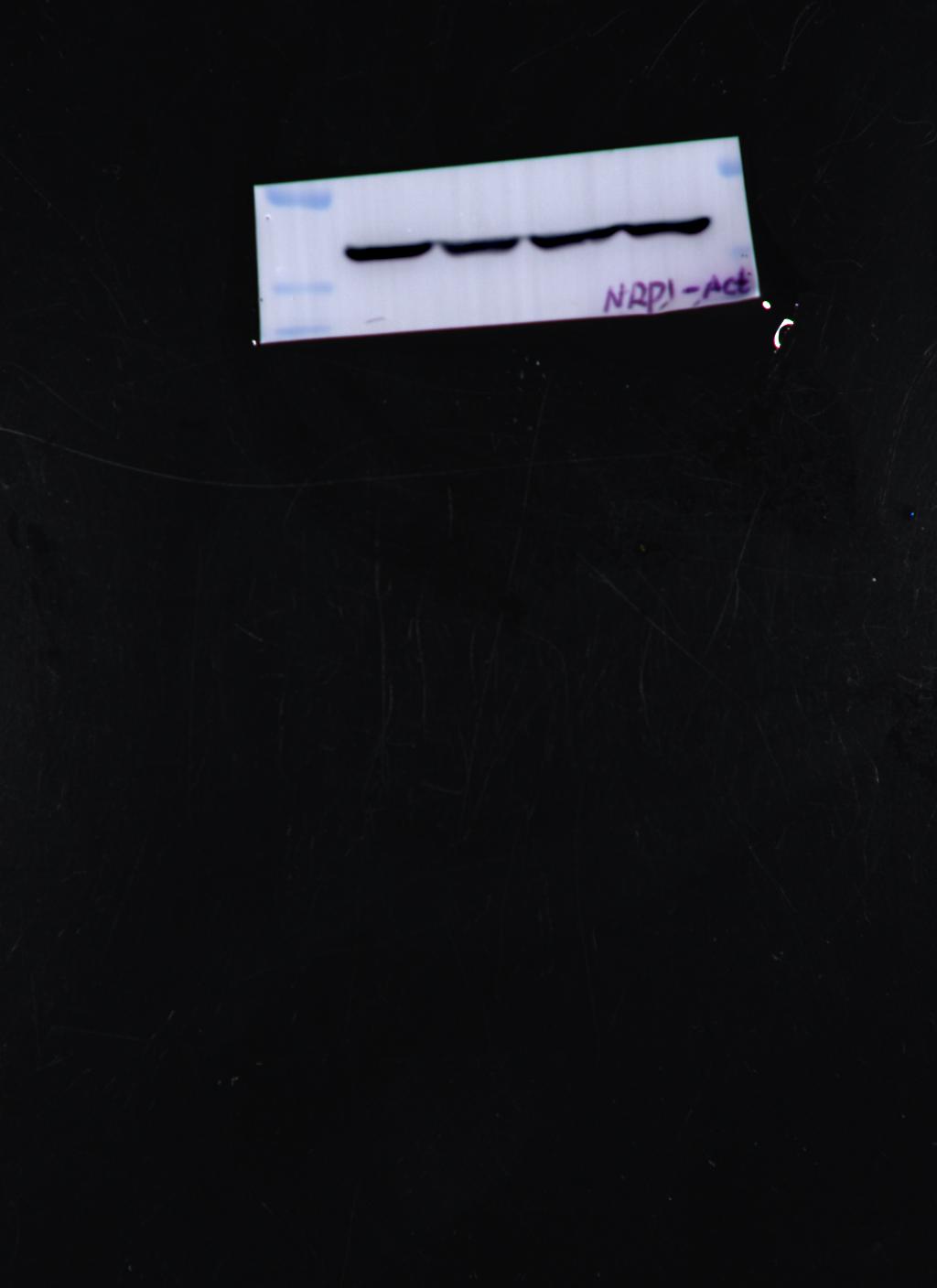

Supplement: Figure 1—source data 1. [file elife-85985-fig1-data1.zip › Figure 1-source data 1/anti-Actin.jpg]

**Fig. 1A**

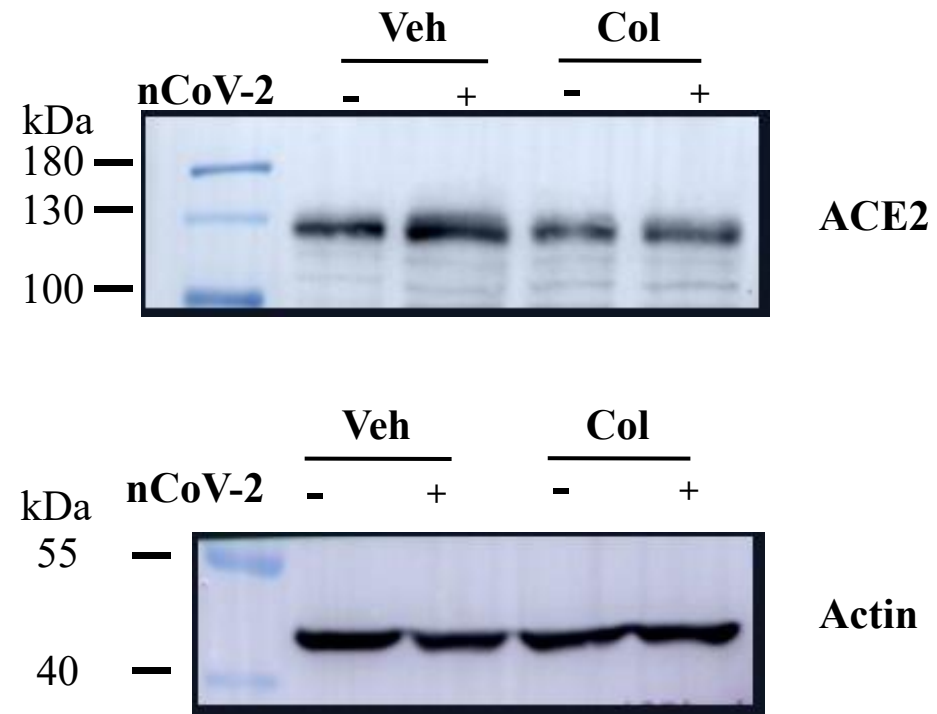

**Full scans for Figure 1A.**

Supplement: Figure 1—source data 2. [file elife-85985-fig1-data2.zip › Figure 1-source data 2/Figure 1A.pdf]

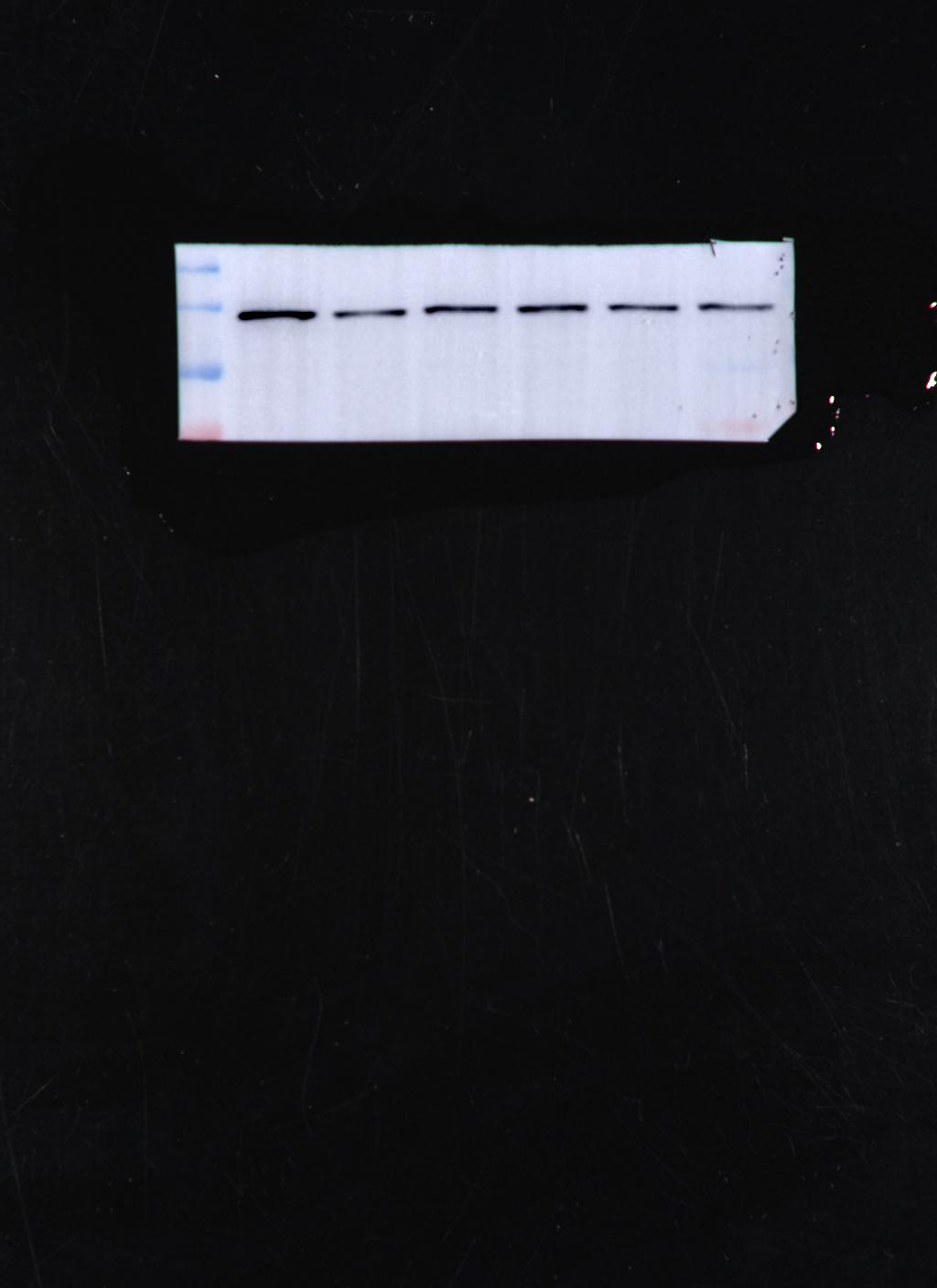

Supplement: Figure 3—source data 1. [file elife-85985-fig3-data1.zip › Figure 3-source data 1/anti-ACE2.jpg]

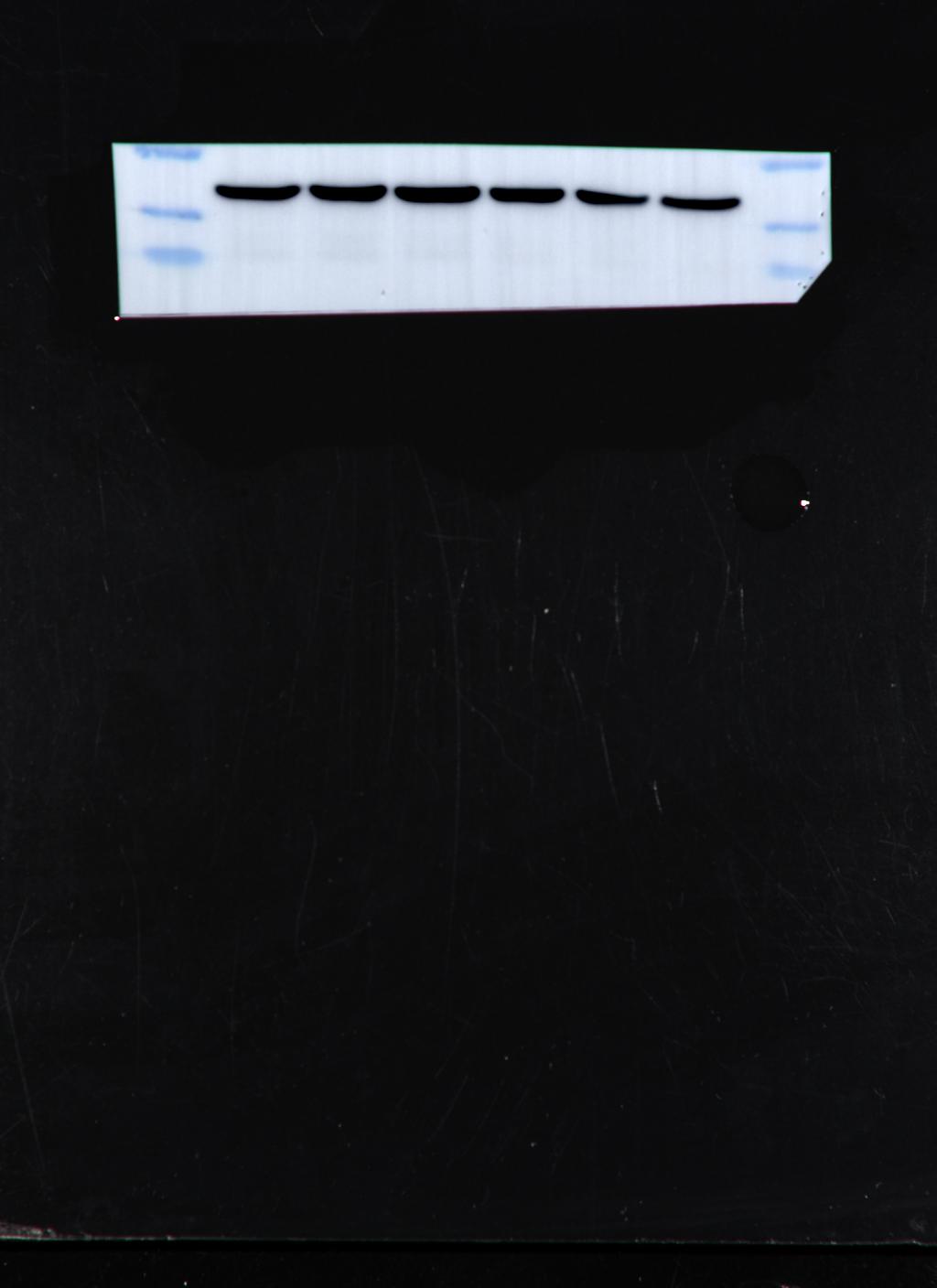

Supplement: Figure 3—source data 1. [file elife-85985-fig3-data1.zip › Figure 3-source data 1/anti-Actin1.jpg]

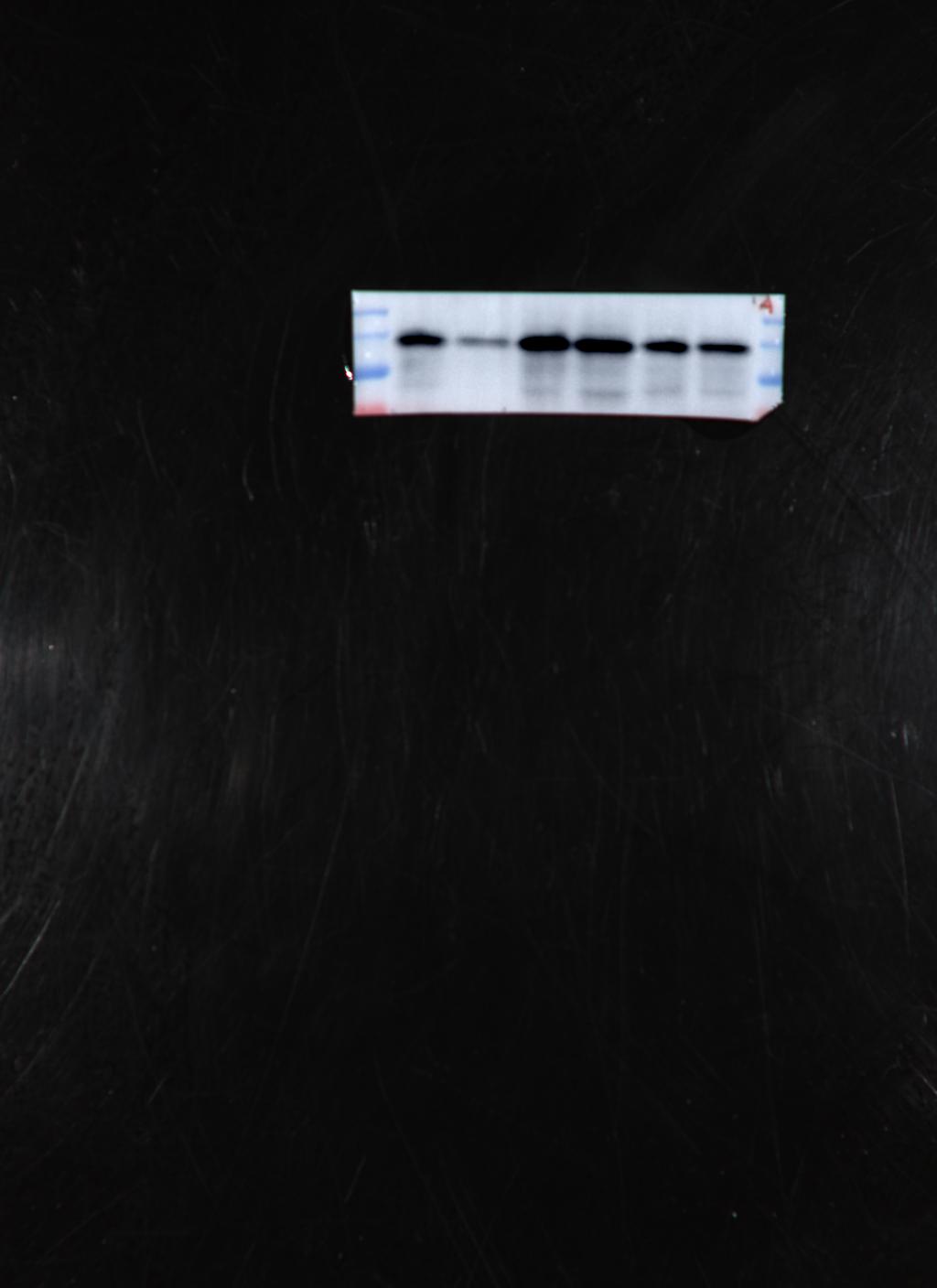

Supplement: Figure 3—source data 2. [file elife-85985-fig3-data2.zip › Figure 3-source data 2/Anti-ACE2.jpg]

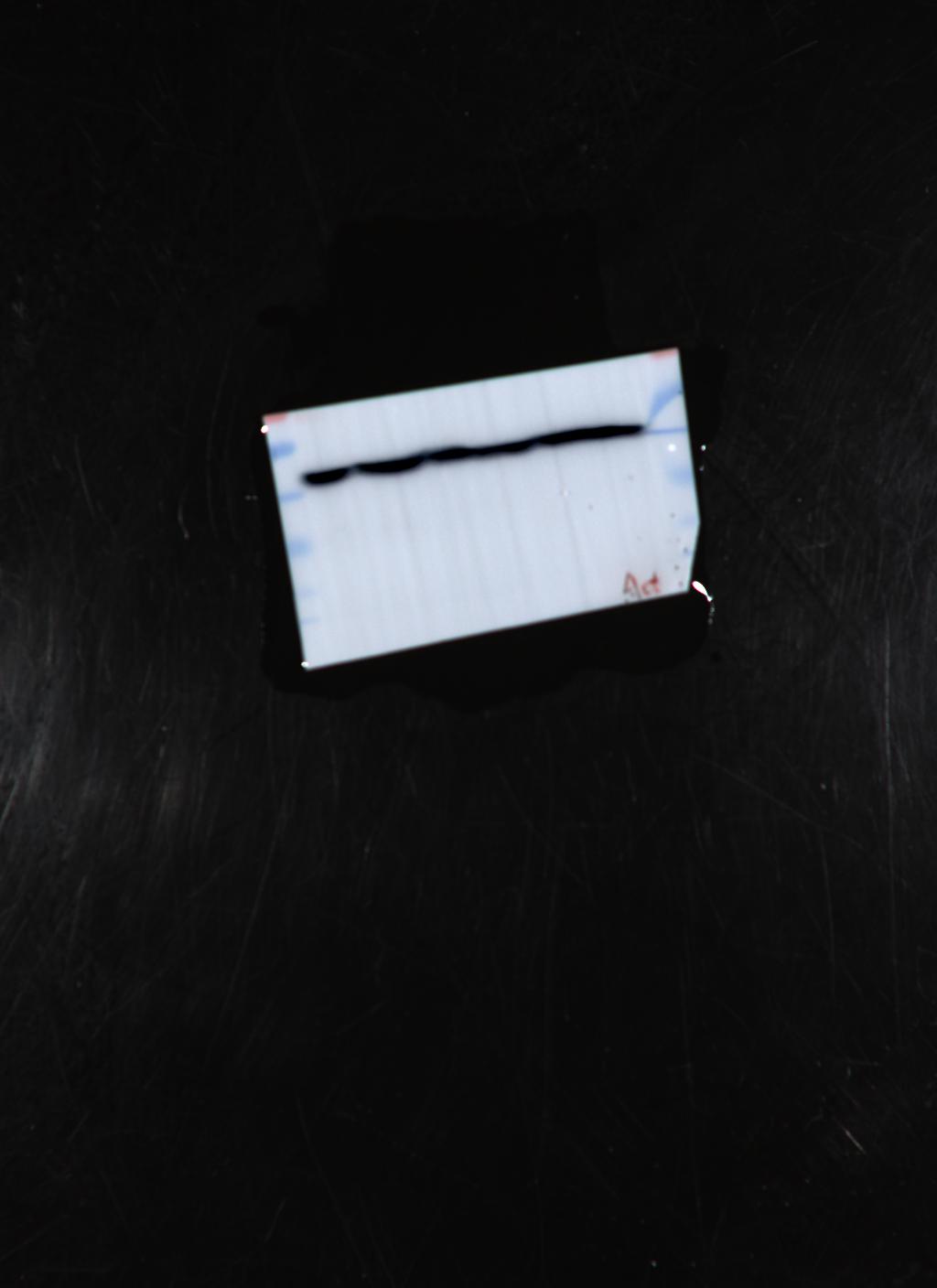

Supplement: Figure 3—source data 2. [file elife-85985-fig3-data2.zip › Figure 3-source data 2/Anti-Actin.jpg]

Fig. 3A

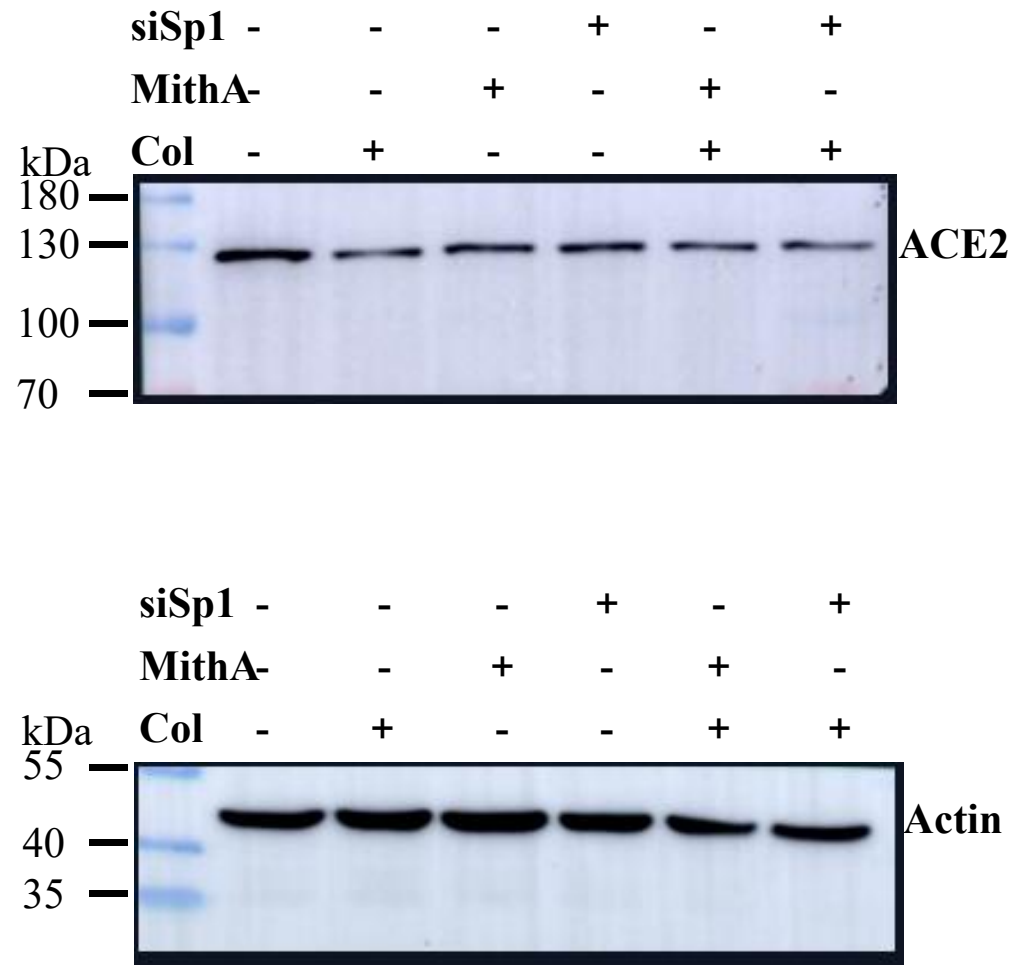

Fig. 3C

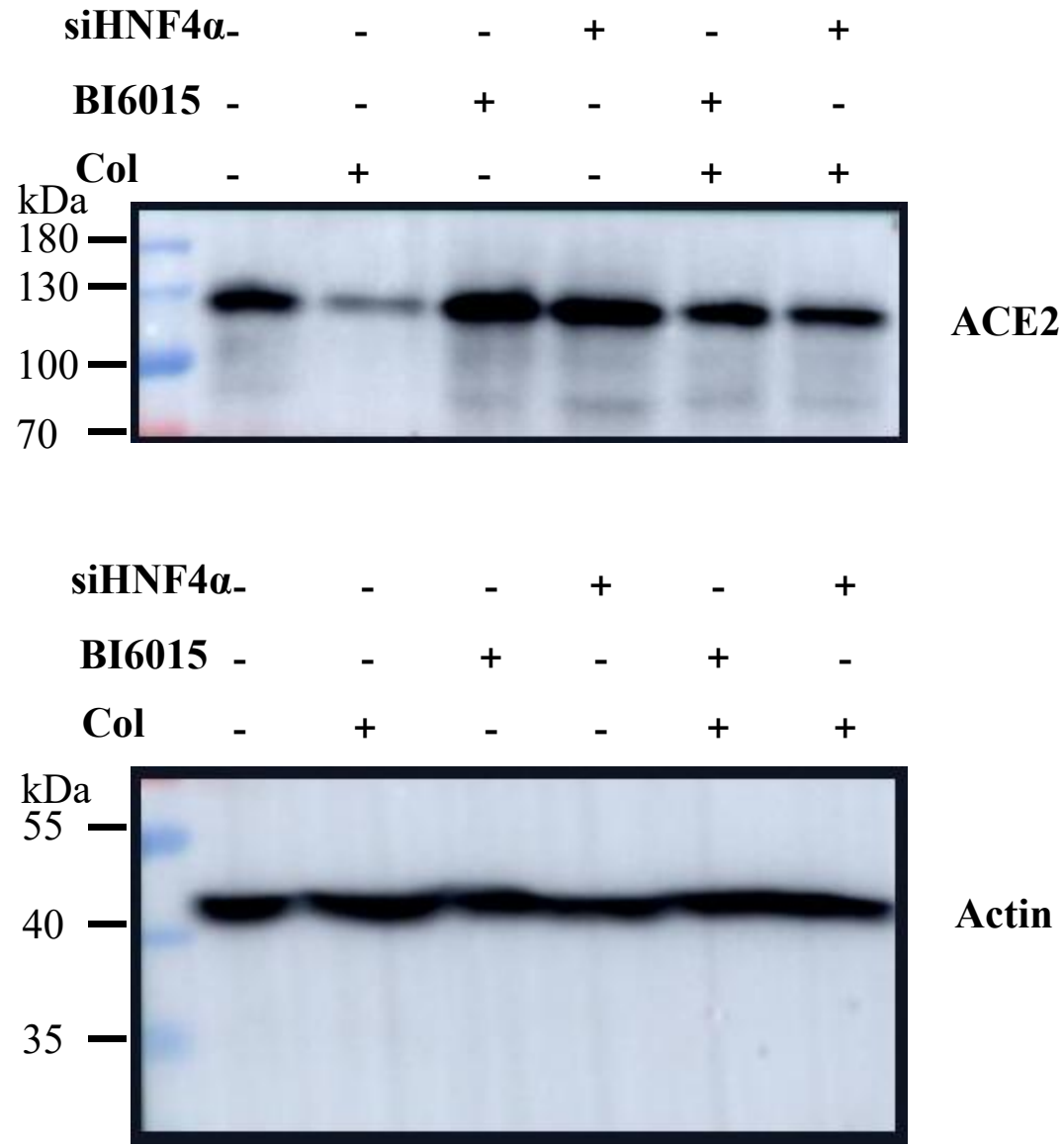

Full scans for Figure 3A and C.

Supplement: Figure 3—source data 3. [file elife-85985-fig3-data3.zip › Figure 3-source data 3/Figure 3A and 3C.pdf]

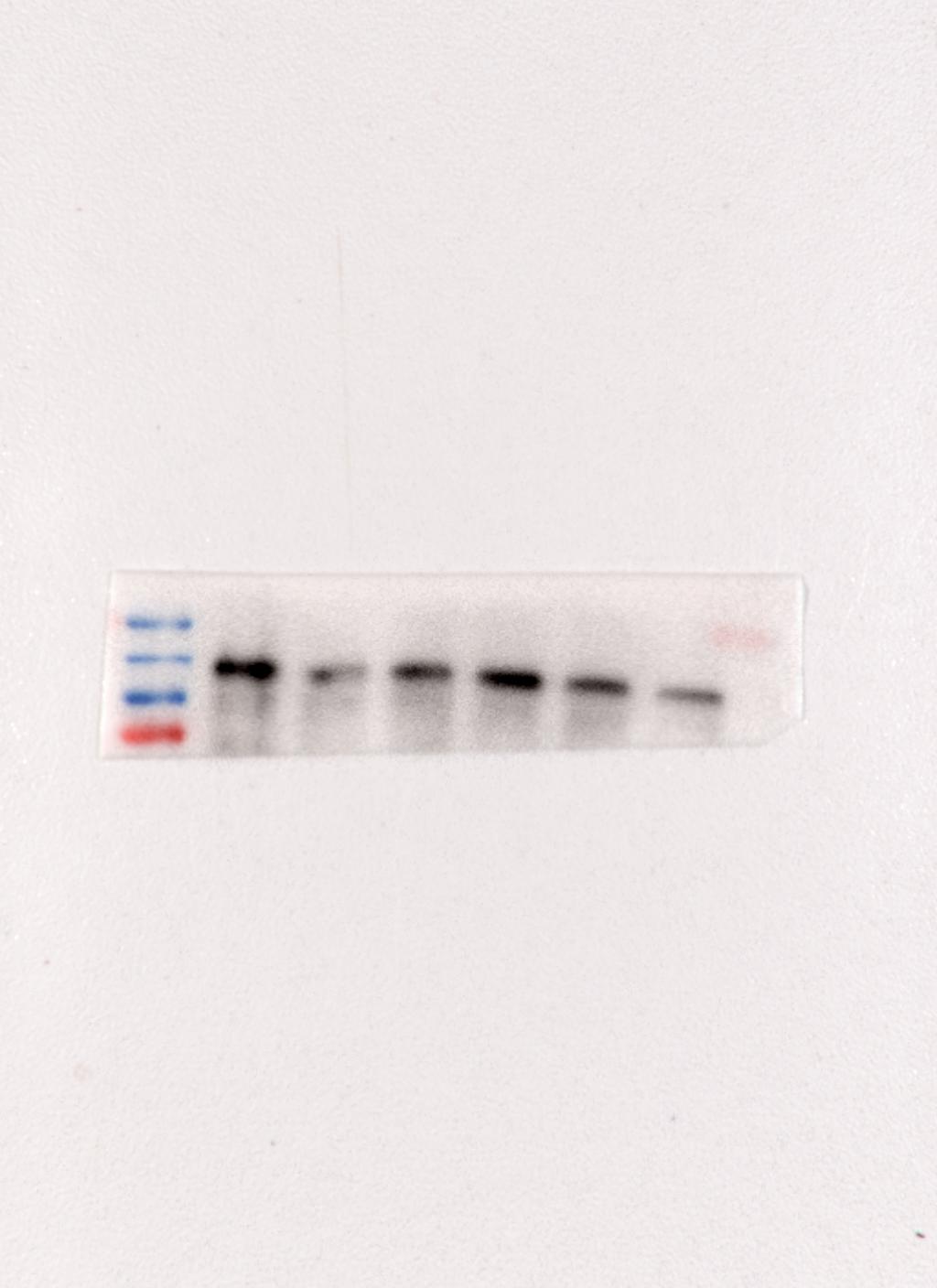

Supplement: Figure 3—figure supplement 2—source data 1. [file elife-85985-fig3-figsupp2-data1.zip › Figure 3-figure supplement 2-source data 1/anti-ACE2.jpg]

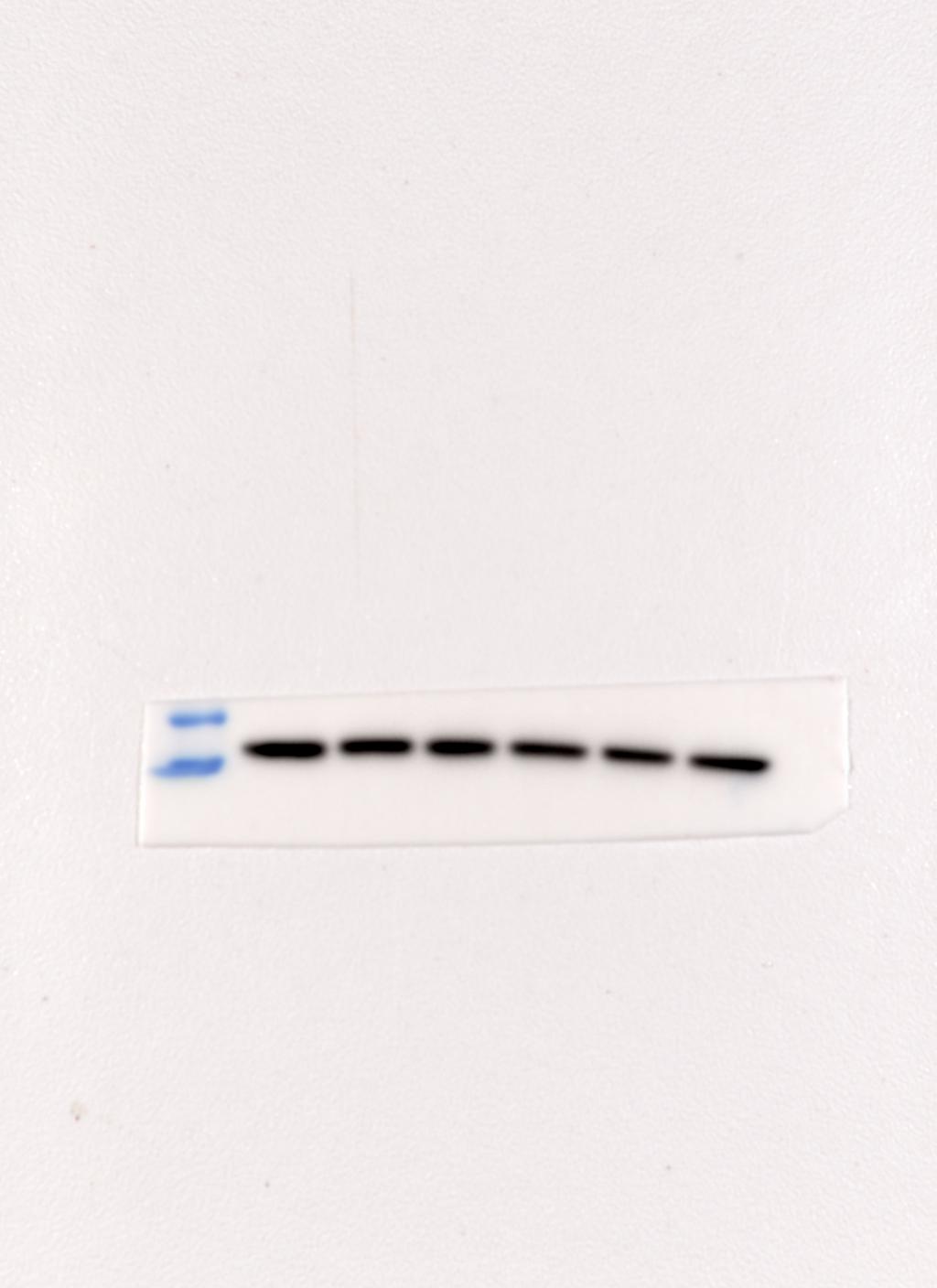

Supplement: Figure 3—figure supplement 2—source data 1. [file elife-85985-fig3-figsupp2-data1.zip › Figure 3-figure supplement 2-source data 1/anti-Actin.jpg]

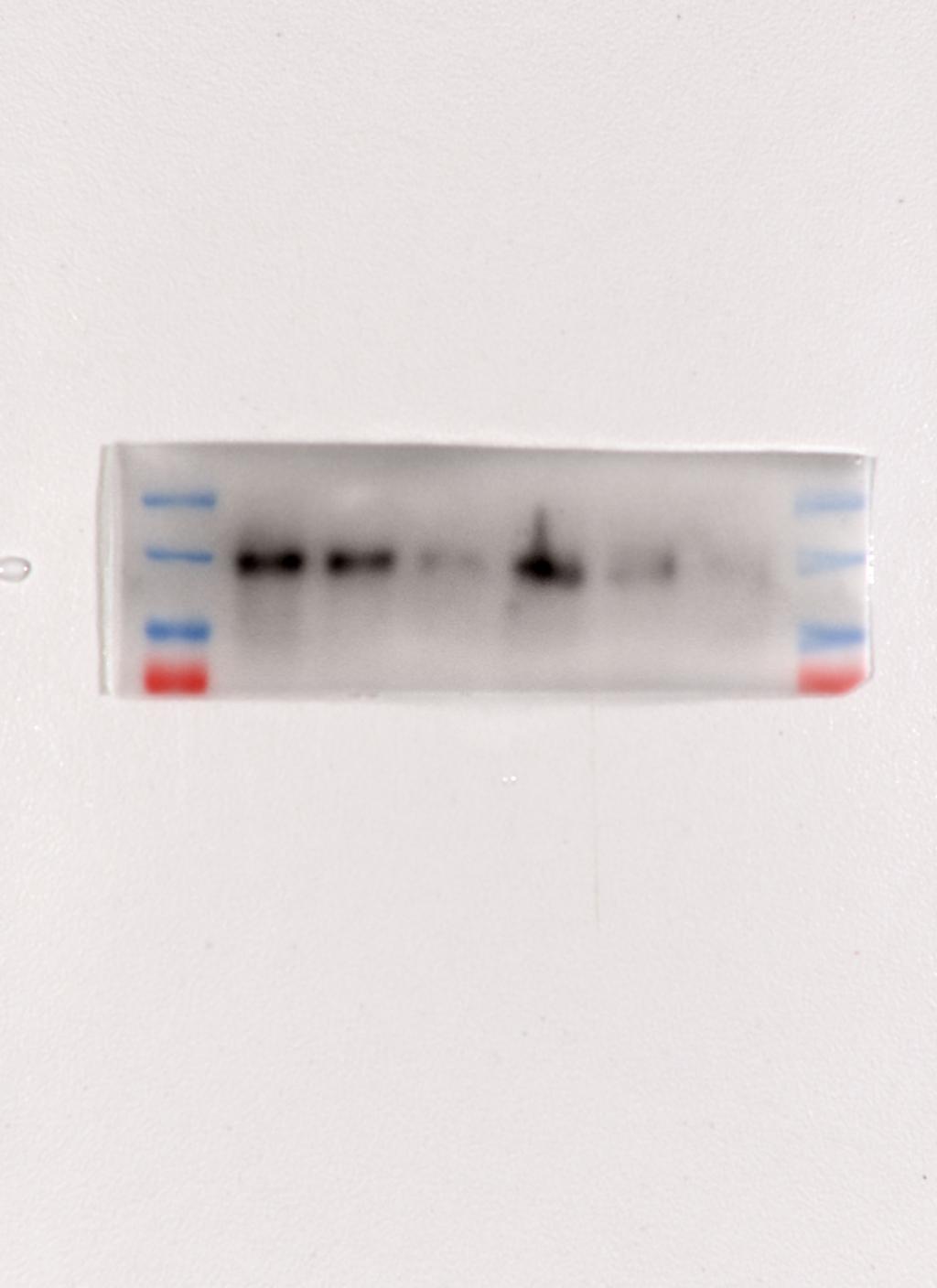

Supplement: Figure 3—figure supplement 2—source data 2. [file elife-85985-fig3-figsupp2-data2.zip › Figure 3-figure supplement 2-source data 2/anti-ACE2.jpg]

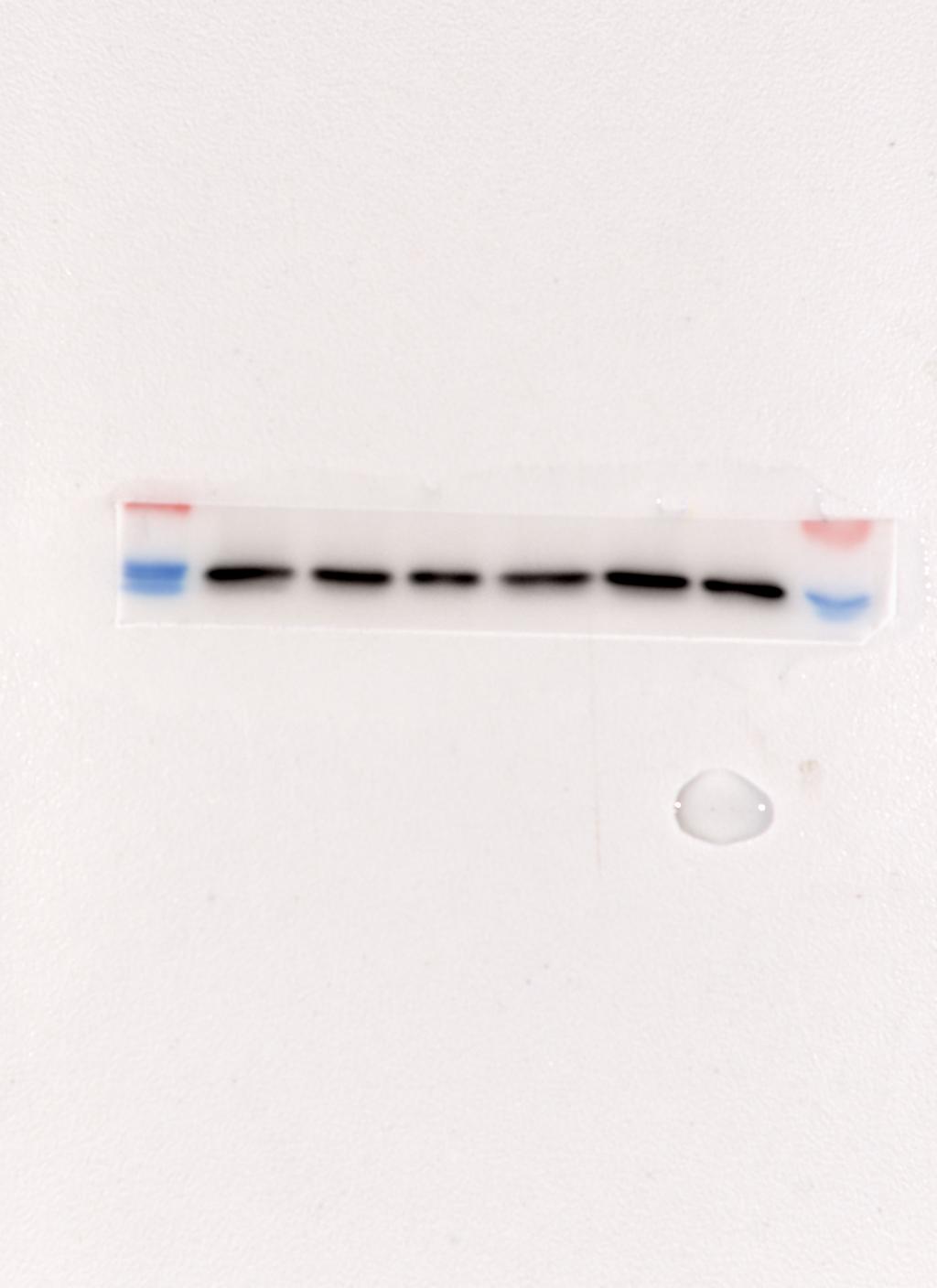

Supplement: Figure 3—figure supplement 2—source data 2. [file elife-85985-fig3-figsupp2-data2.zip › Figure 3-figure supplement 2-source data 2/anti-Actin.jpg]

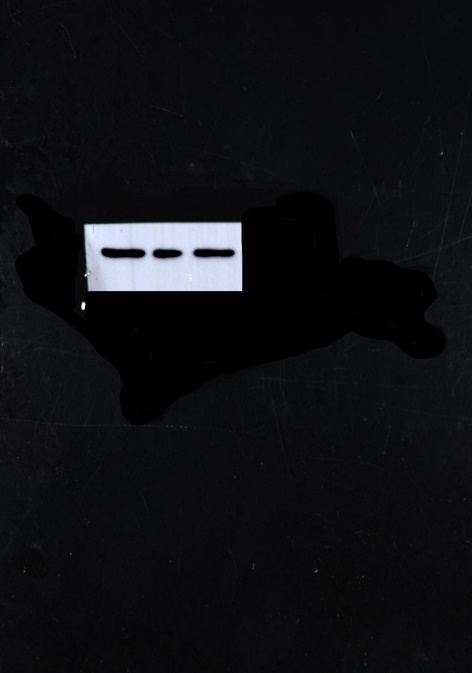

Supplement: Figure 4—source data 3. [file elife-85985-fig4-data3.zip › Figure 4-source data 3/anti-Actin.jpg]

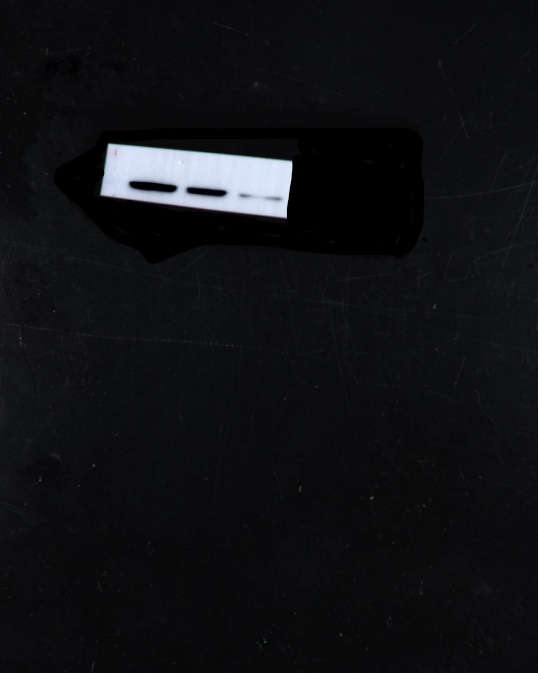

Supplement: Figure 4—source data 3. [file elife-85985-fig4-data3.zip › Figure 4-source data 3/anti-HNF4α.jpg]

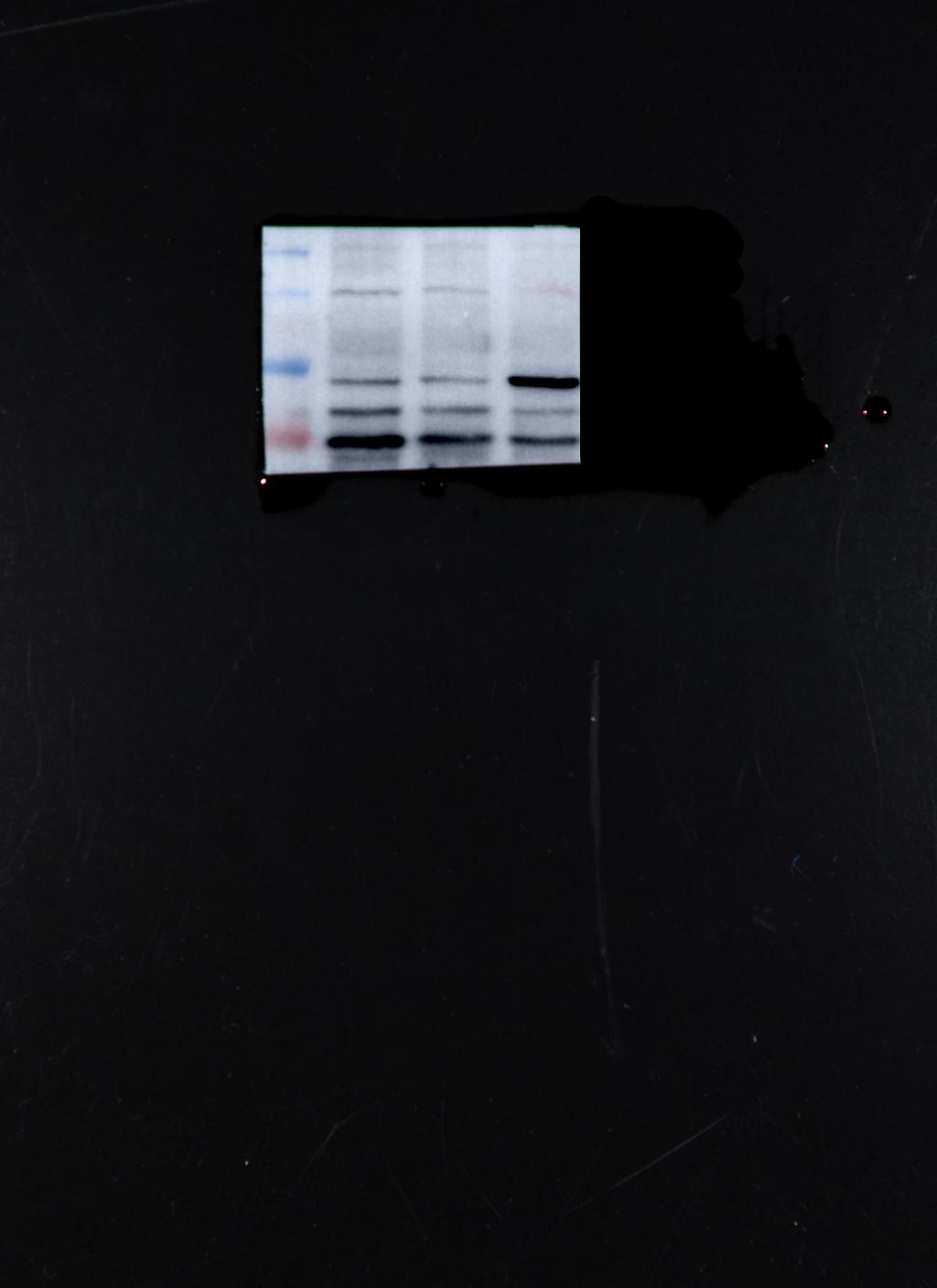

Supplement: Figure 4—source data 3. [file elife-85985-fig4-data3.zip › Figure 4-source data 3/anti-psp1.jpg]

**Fig. 4E**

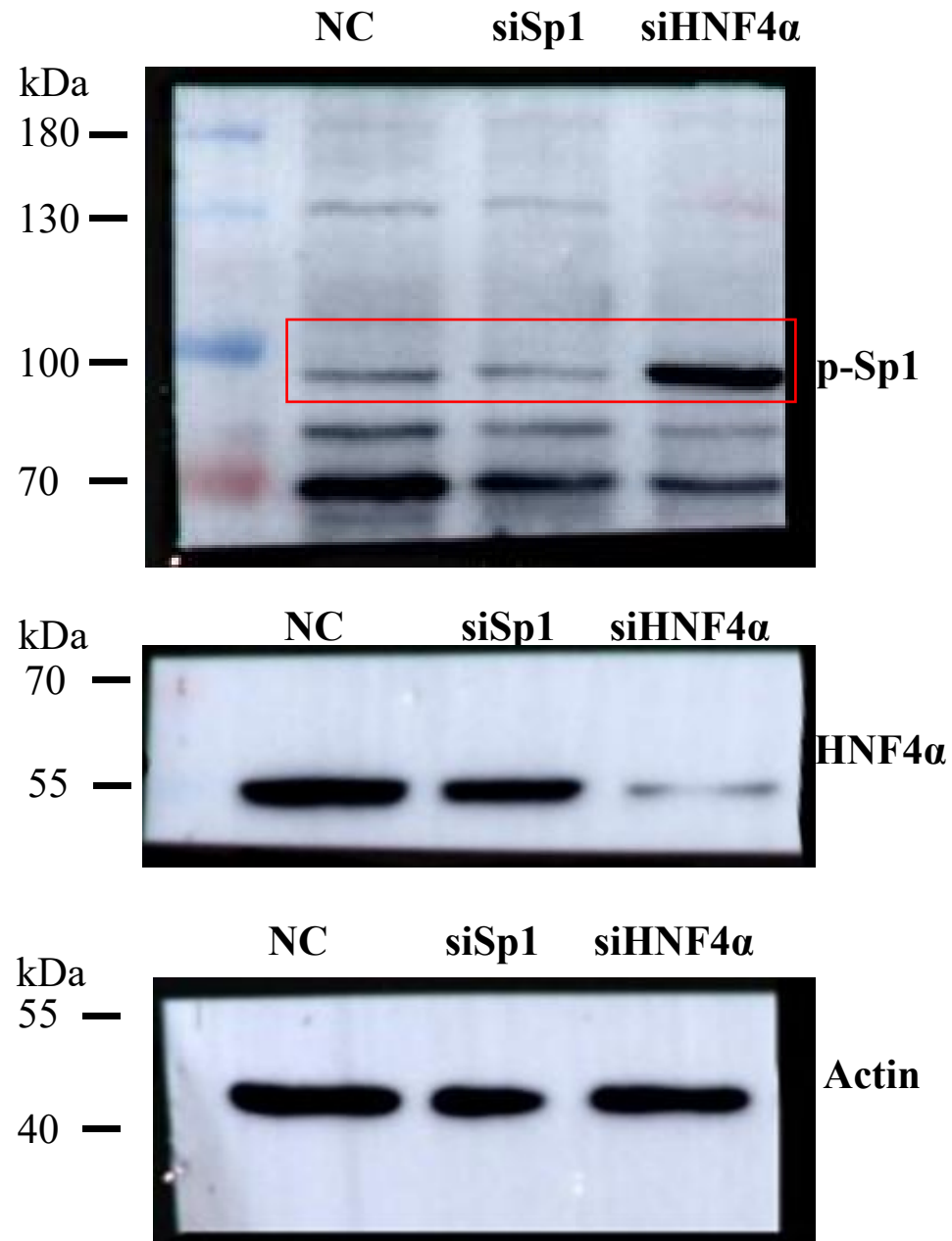

**Fig. 4G**

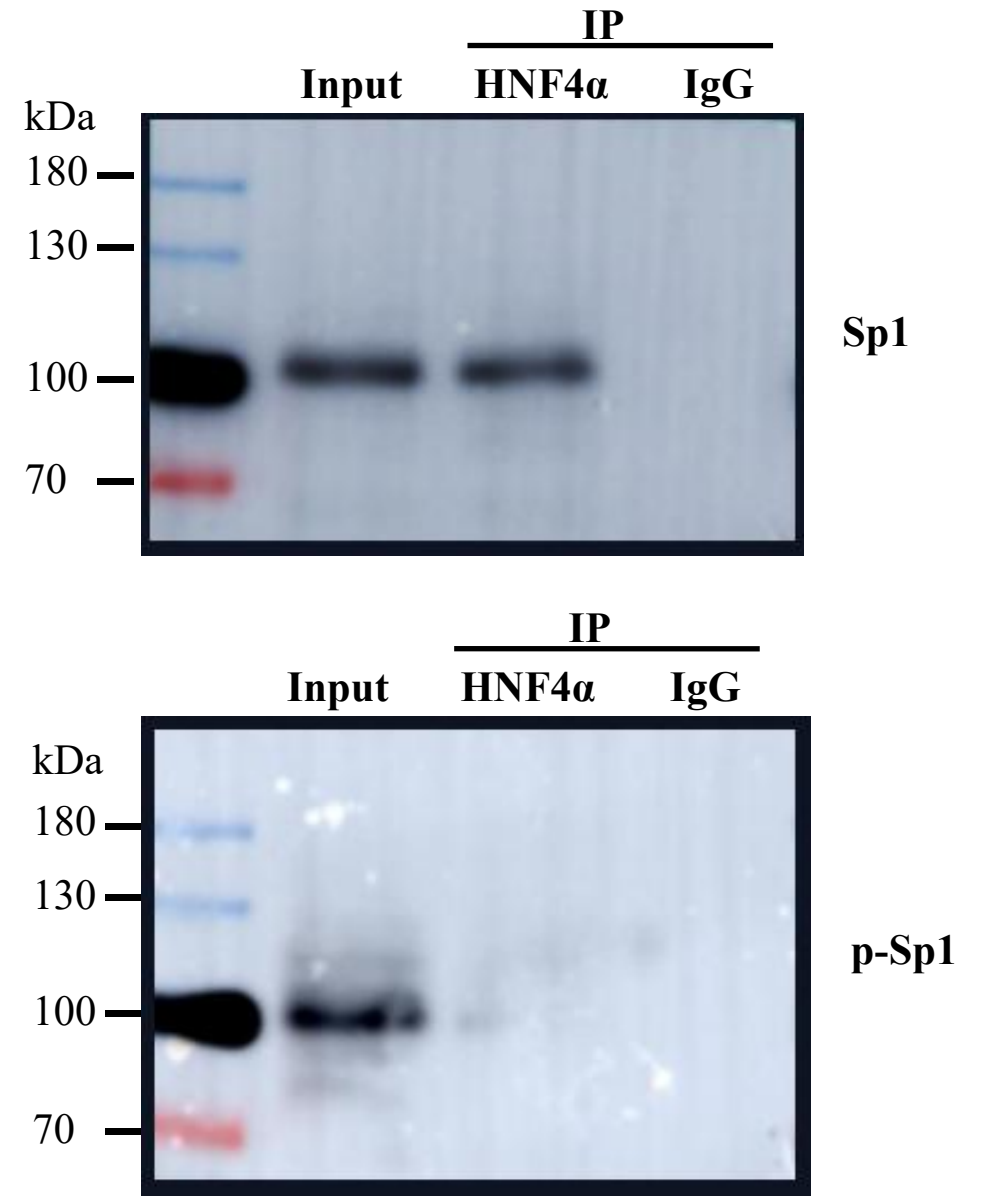

**Full scans for Figure 4E and G.**

Supplement: Figure 4—source data 4. [file elife-85985-fig4-data4.zip › Figure 4-source data 4/Figure 4E and 4G.pdf]

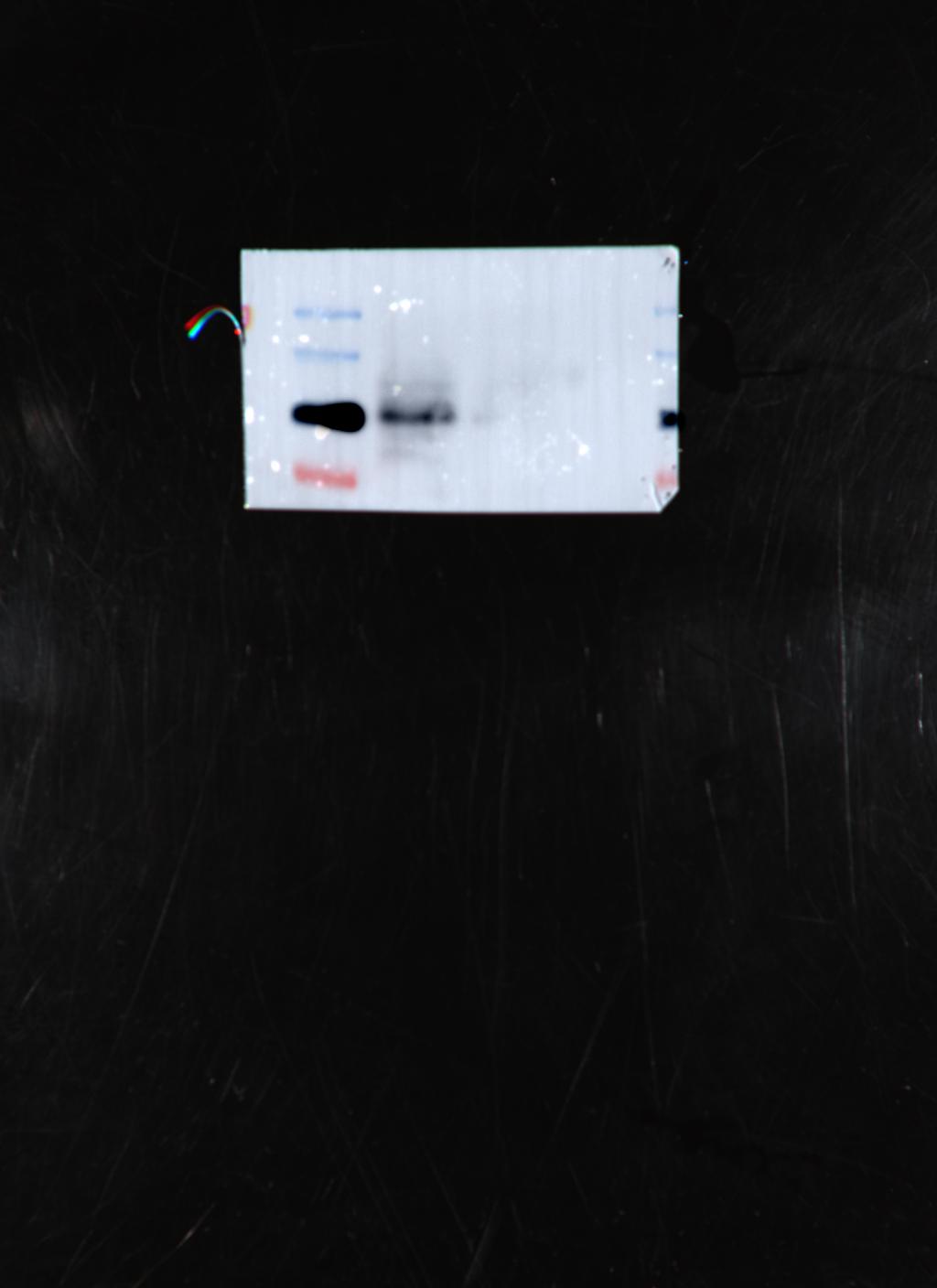

Supplement: Figure 4—source data 6. [file elife-85985-fig4-data6.zip › Figure 4-source data 6/anti-p-Sp1.jpg]

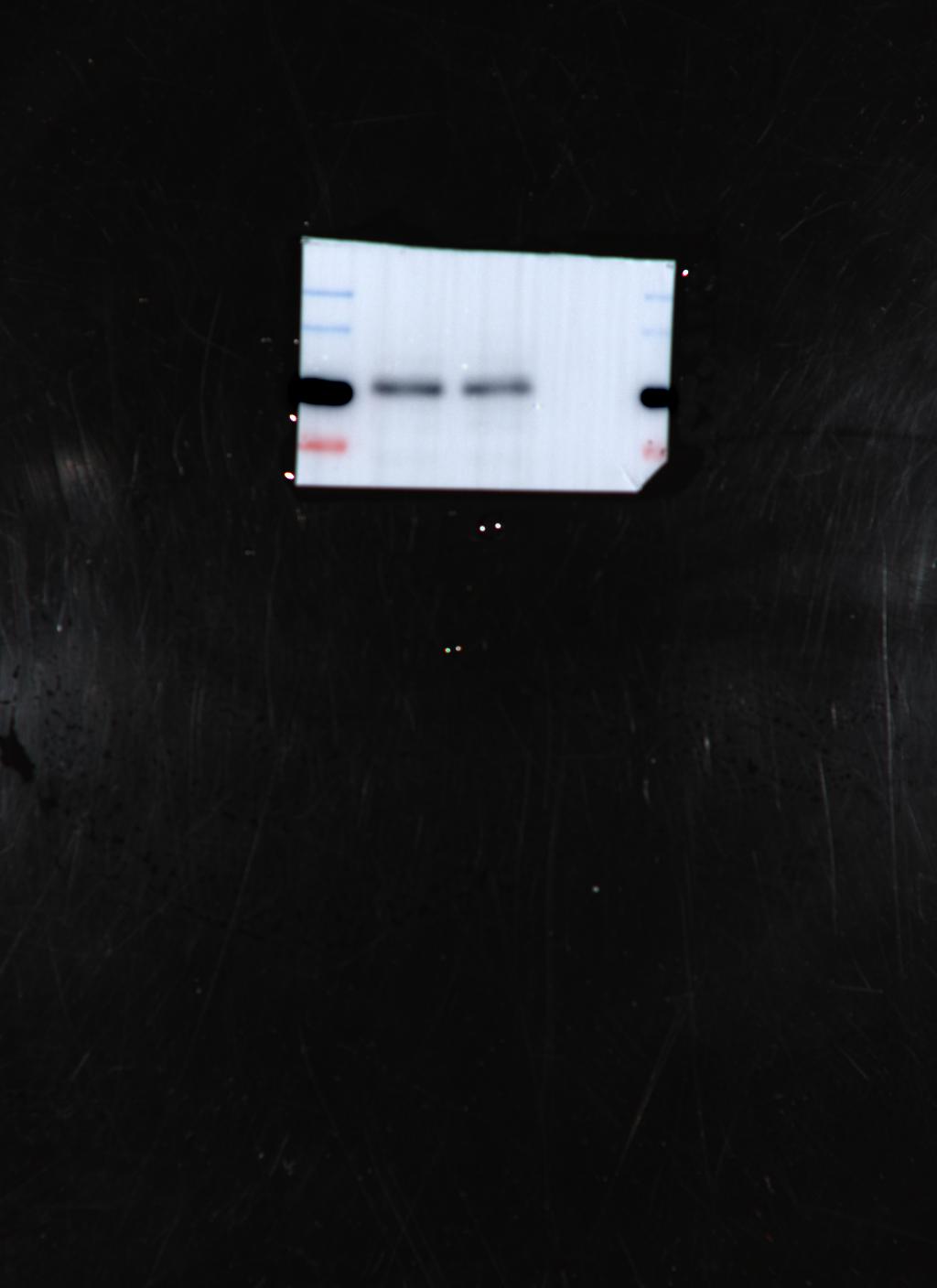

Supplement: Figure 4—source data 6. [file elife-85985-fig4-data6.zip › Figure 4-source data 6/anti-Sp1.jpg]

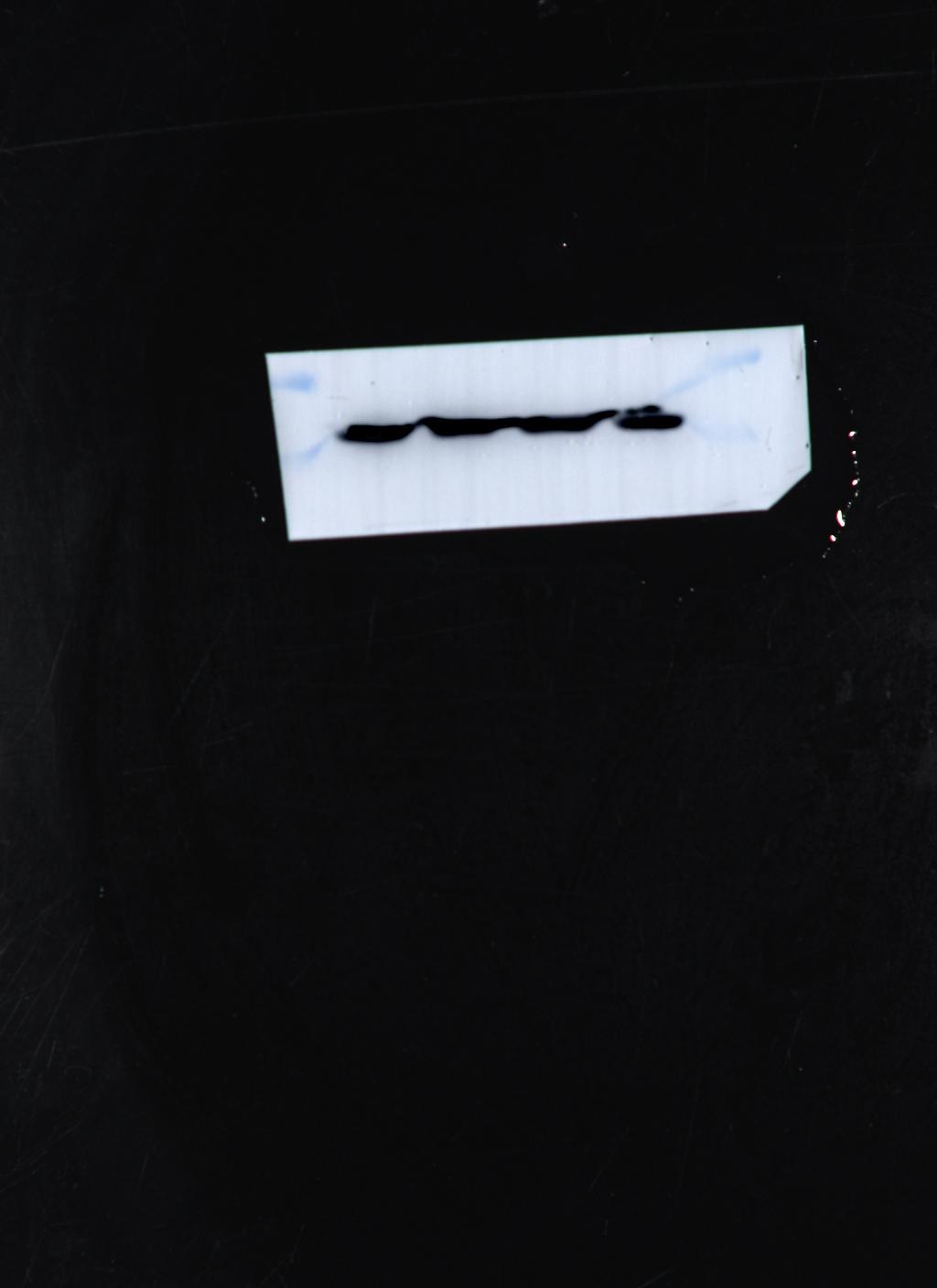

Supplement: Figure 5—source data 1. [file elife-85985-fig5-data1.zip › Figure 5-source data 1/anti-actin.jpg]

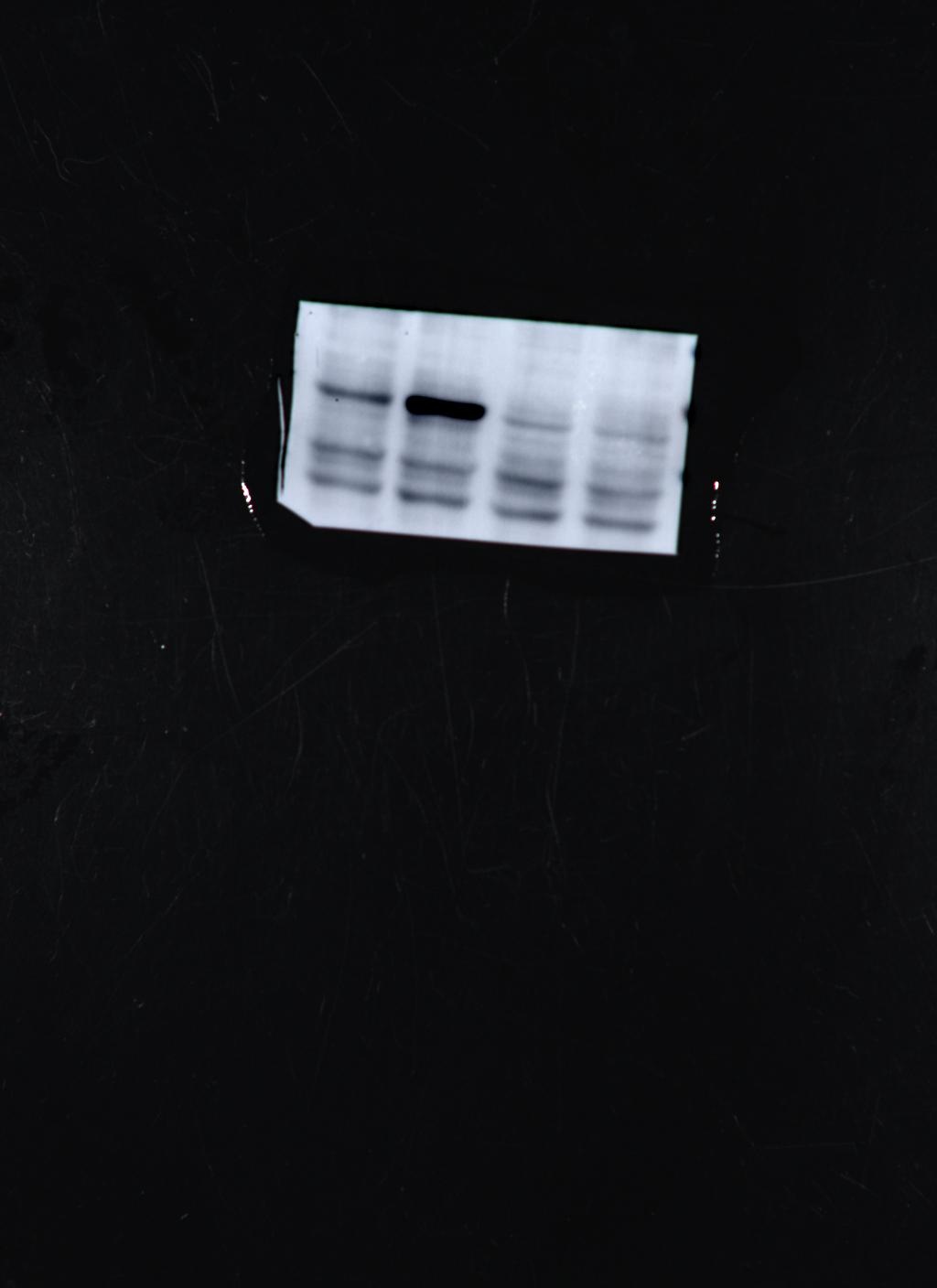

Supplement: Figure 5—source data 1. [file elife-85985-fig5-data1.zip › Figure 5-source data 1/anti-AKT (473).jpg]

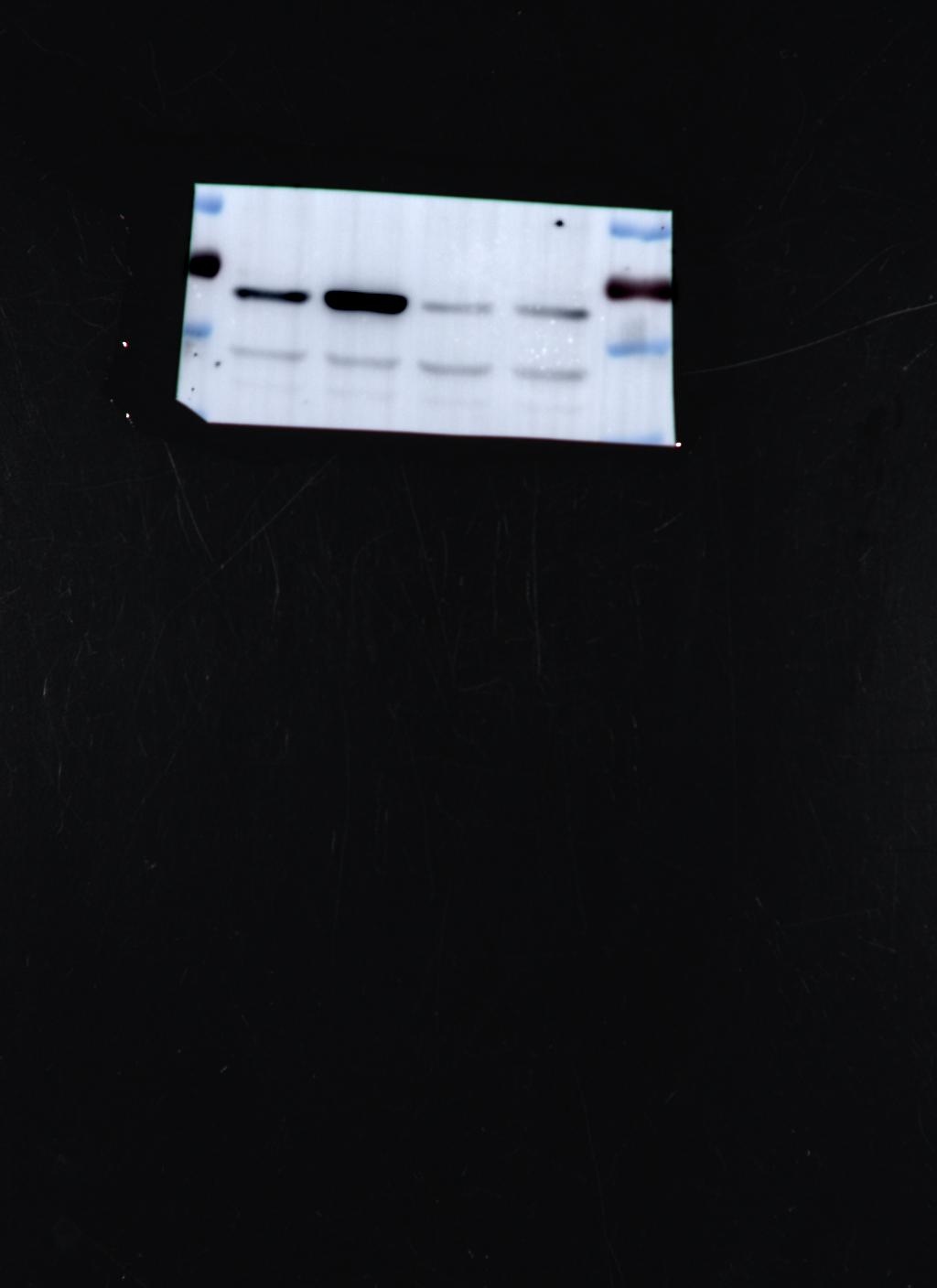

Supplement: Figure 5—source data 1. [file elife-85985-fig5-data1.zip › Figure 5-source data 1/anti-AKT (T308).jpg]

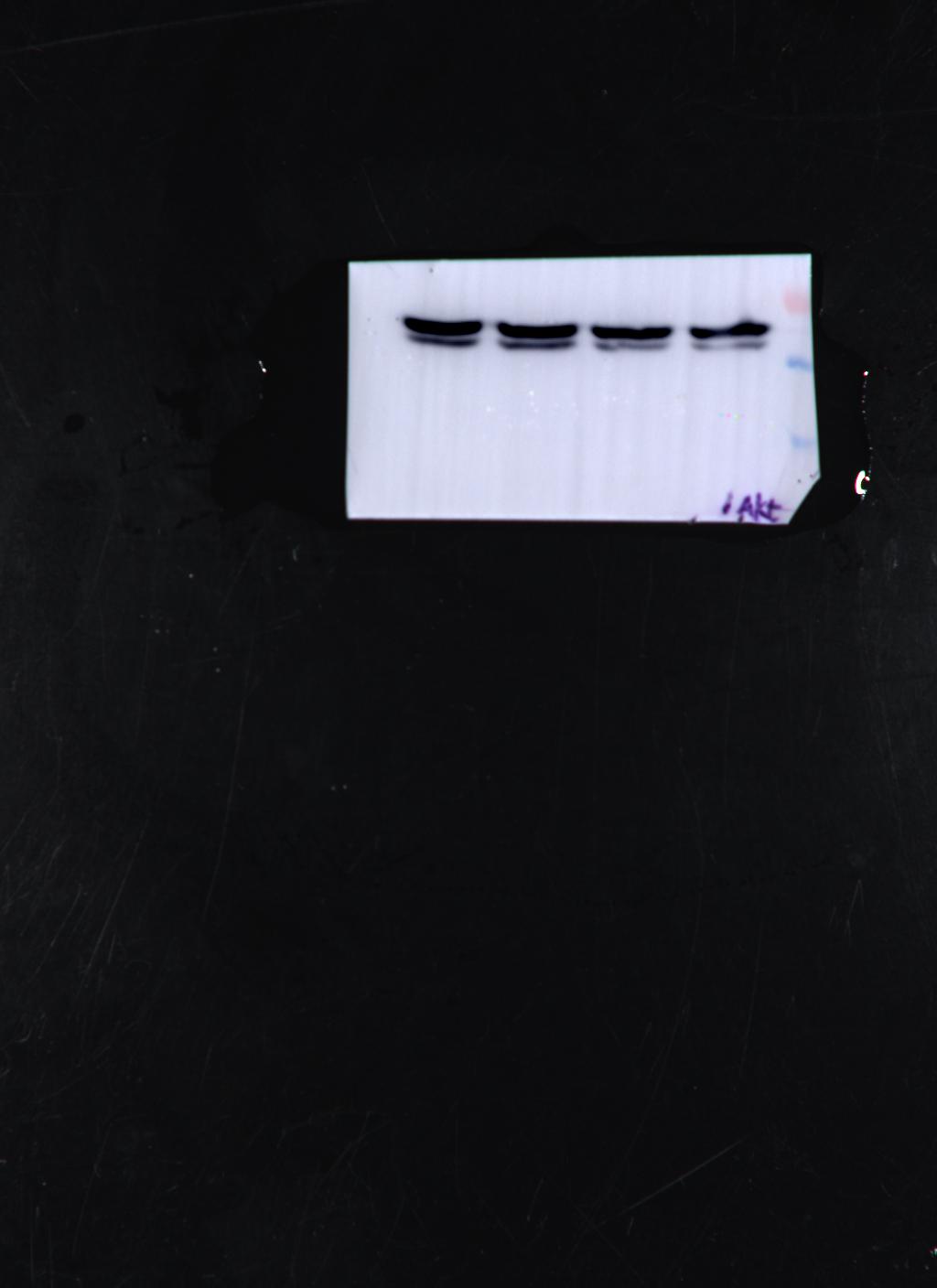

Supplement: Figure 5—source data 1. [file elife-85985-fig5-data1.zip › Figure 5-source data 1/anti-panAKT.jpg]

**Fig. 5A**

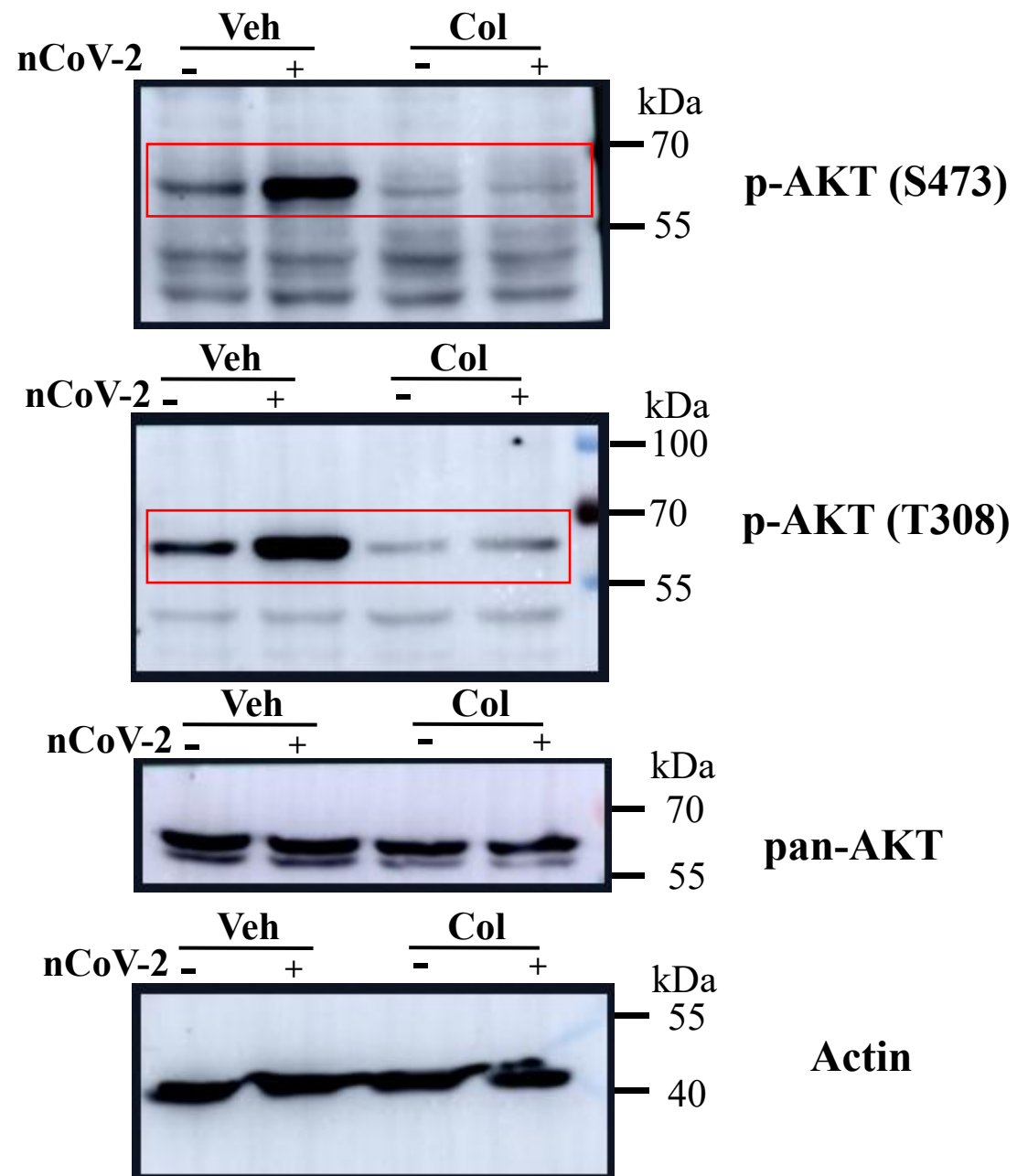

Full scans for Figure 5A and D.

**Fig. 5D**

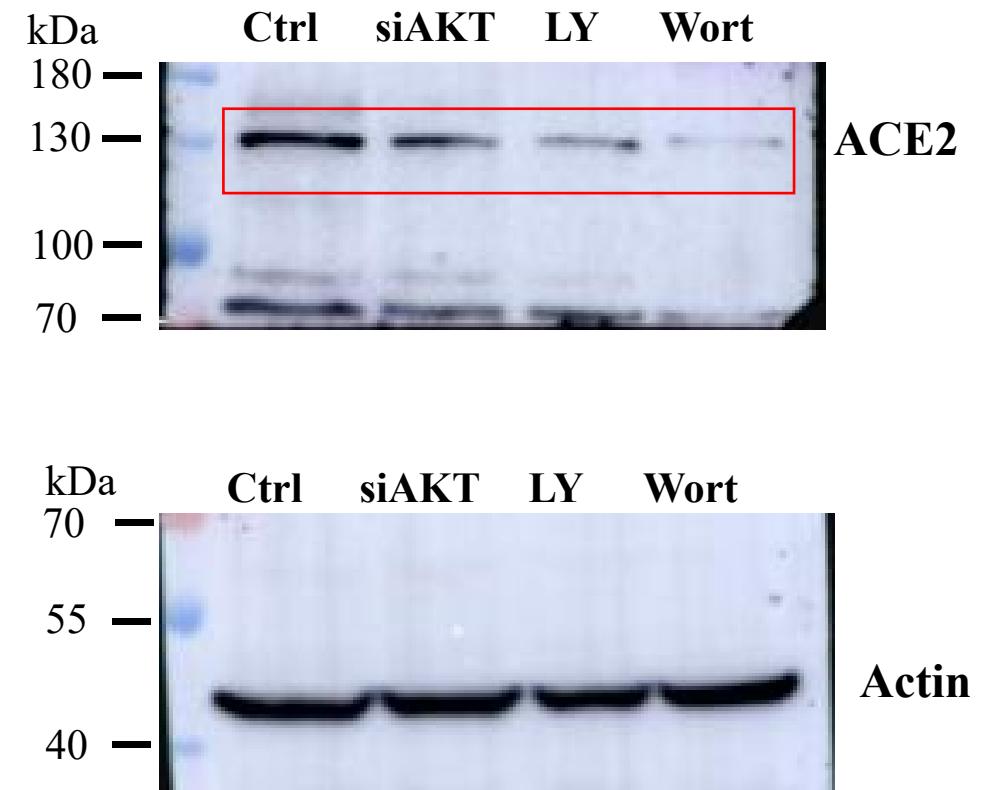

Supplement: Figure 5—source data 2. [file elife-85985-fig5-data2.zip › Figure 5-source data 2/Figure 5A and 5D.pdf]

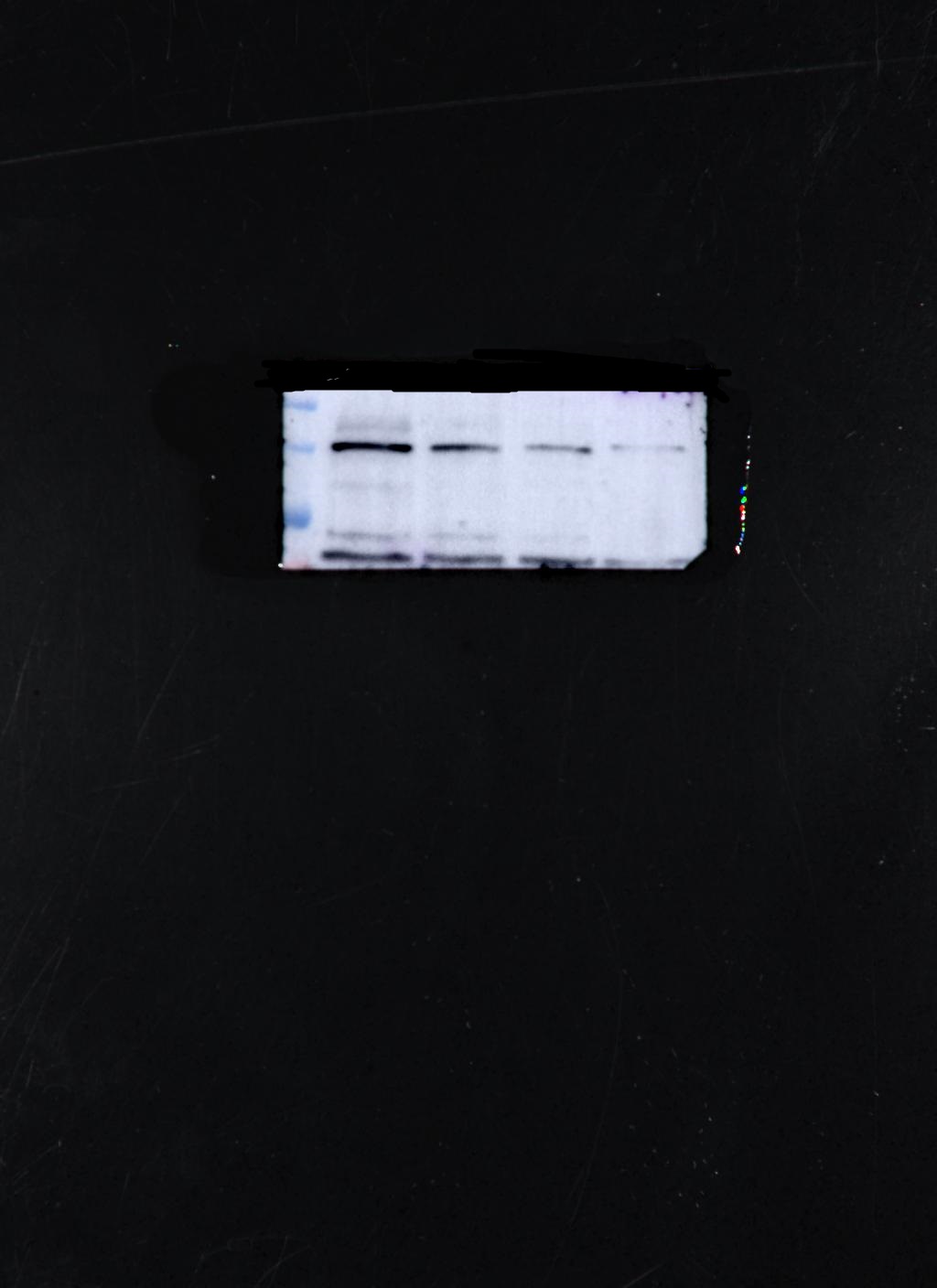

Supplement: Figure 5—source data 4. [file elife-85985-fig5-data4.zip › Figure 5-source data 4/anti-ACE2.jpg]

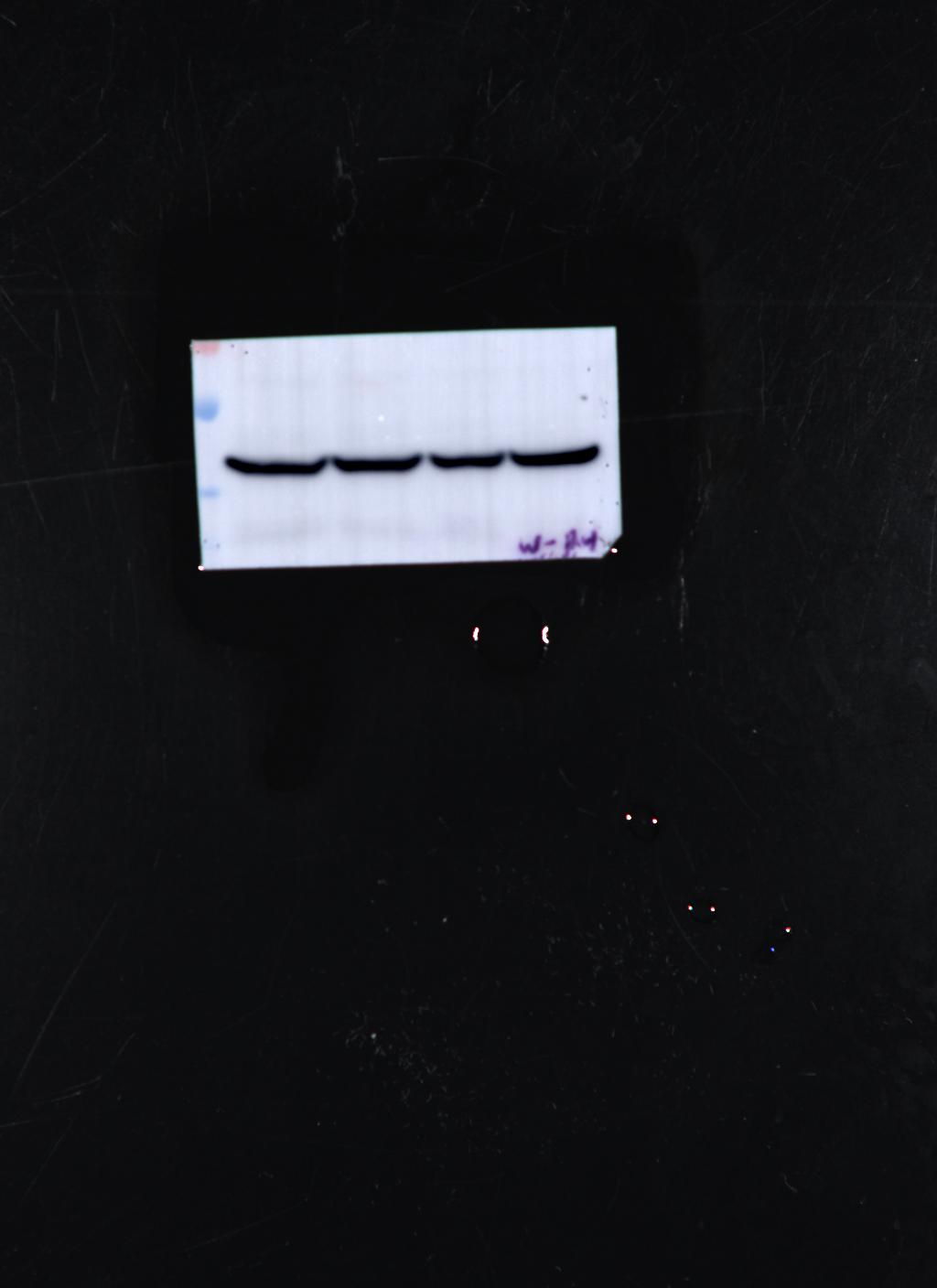

Supplement: Figure 5—source data 4. [file elife-85985-fig5-data4.zip › Figure 5-source data 4/anti-Actin.jpg]
