# Supplementary material for: Transcriptional regulation of SARS-CoV-2 receptor ACE2 by SP1
Source: eLife. 2024 Feb 20;13:e85985. doi: 10.7554/eLife.85985 (PMC10878691; doi:10.7554/eLife.85985)
Supplement: Figure 3—figure supplement 2—source data 3. [file elife-85985-fig3-figsupp2-data3.zip › Figure 3-figure supplement 2-source data 3/Figure 3-figure supplement 2A and 2C.pdf]

Figure 3-figure supplement 2A

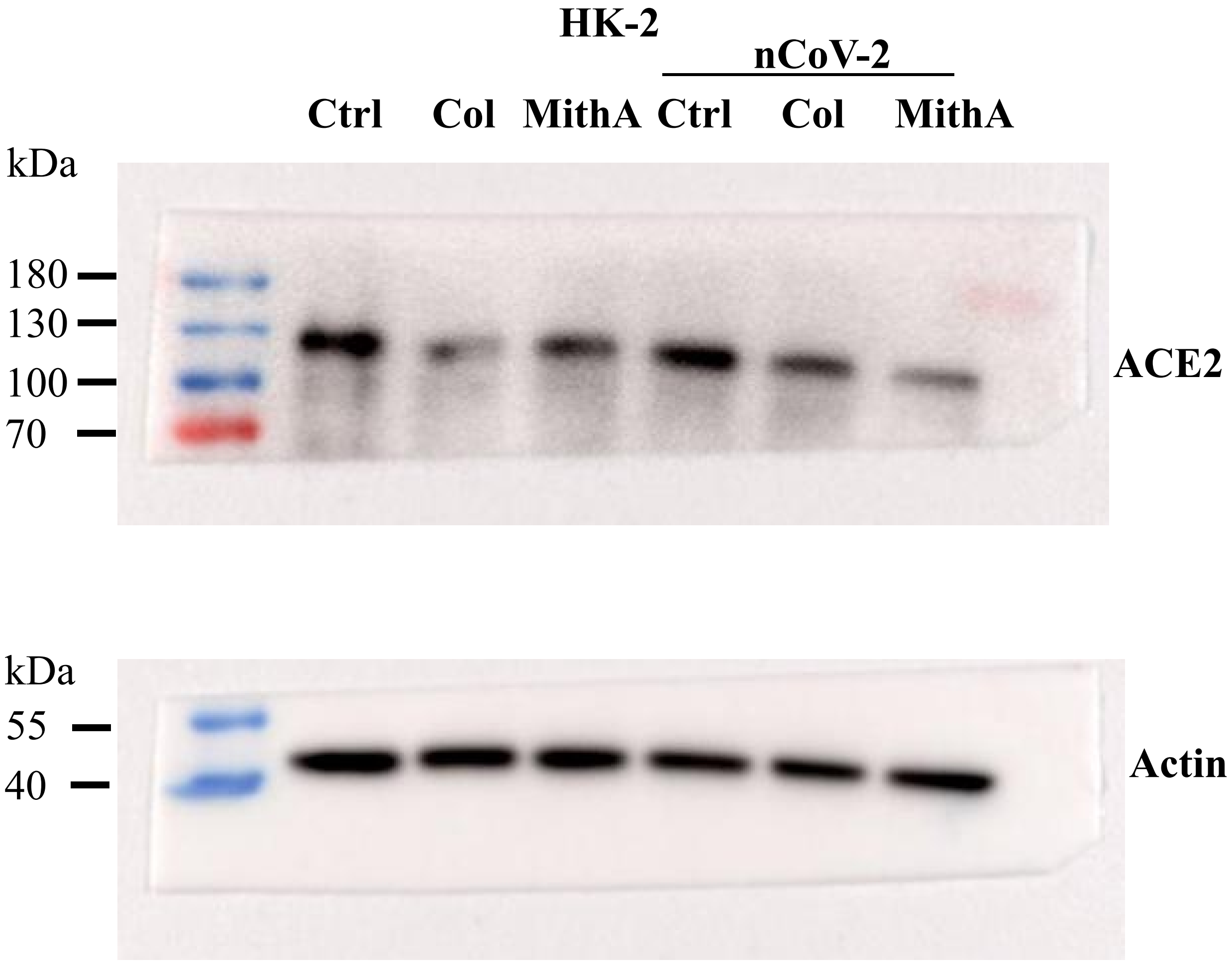

Figure 3-figure supplement 2C

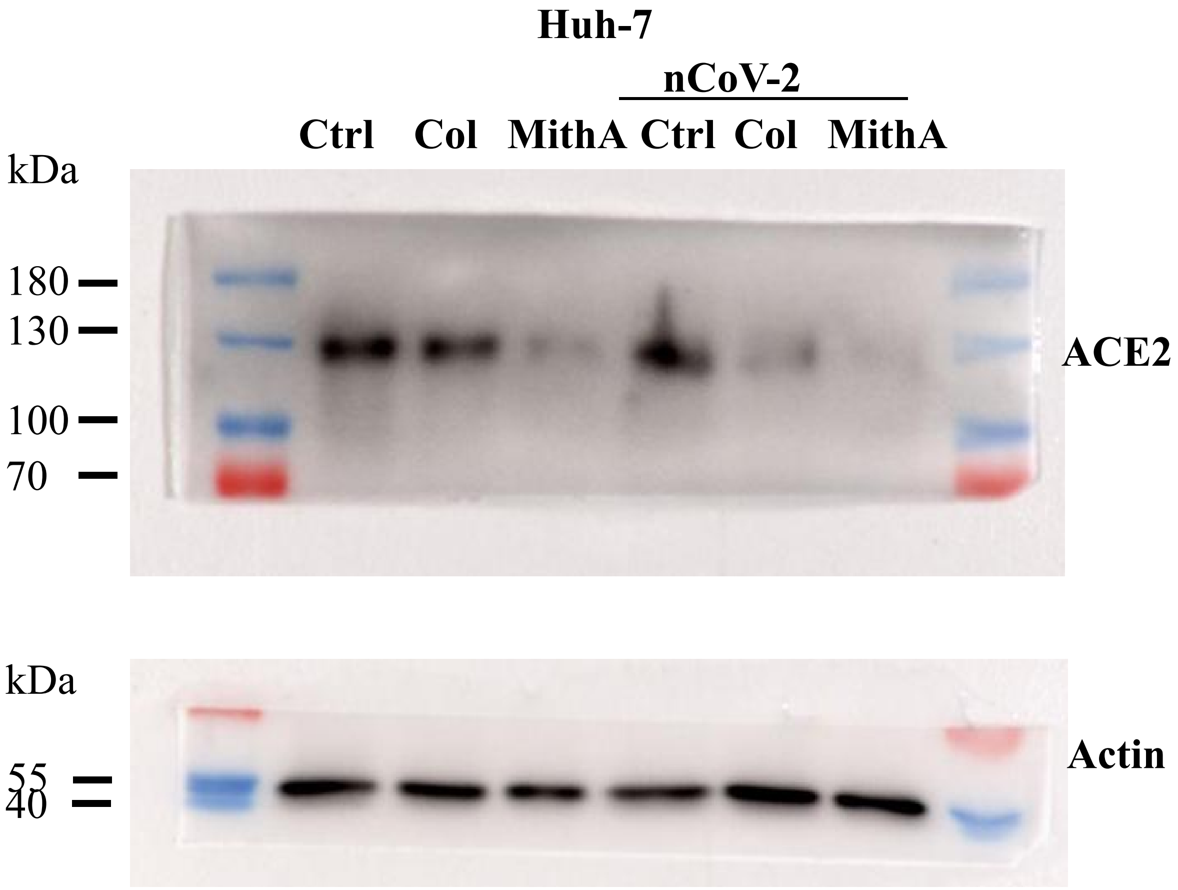

Full scans for Figure 3-figure supplement 2A and 2C.
